# Feasibility and acceptability of a digital tool to support community-based screening for COVID-19 and other priority medical conditions across rural and peri-urban communities in Guinea

**DOI:** 10.1093/oodh/oqae044

**Published:** 2024-10-25

**Authors:** Nasser Diallo, Mamadou Bobo, Aboubacar Diallo, Abdoul Karim Baldé, Alpha Oumar Bah, Mamady Kourouma, Mamady Cisse, Nick Banks, Rigveda Kadam, Khairunisa Suleiman, Paula Akugizibwe

**Affiliations:** Clinic+O, Nongo, Commune de Ratoma, Conakry, Guinea; Clinic+O, Nongo, Commune de Ratoma, Conakry, Guinea; Independent Consultant, Unit 4, 294 Castlehill Dr, Blackheath, Johannesburg, 2195 South Africa; Clinic+O, Nongo, Commune de Ratoma, Conakry, Guinea; Clinic+O, Nongo, Commune de Ratoma, Conakry, Guinea; Department of Community Health, Ministry of Health, Blvd de commerce, Conakry, Guinea; Department of Community Health, Ministry of Health, Blvd de commerce, Conakry, Guinea; FIND, Campus Biotech, Chemin des Mines 9, 1202 Geneva, Switzerland; FIND, Campus Biotech, Chemin des Mines 9, 1202 Geneva, Switzerland; FIND, Campus Biotech, Chemin des Mines 9, 1202 Geneva, Switzerland; FIND, Campus Biotech, Chemin des Mines 9, 1202 Geneva, Switzerland

**Keywords:** digital, diagnosis, community-based screening, malaria, NCDs, Guinea

## Abstract

Access to primary healthcare, including diagnostic testing, is limited in Guinea, particularly for low-income residents of rural communities. Here we share findings from an interventional operational research study evaluating the feasibility of deploying a digital tool and rapid diagnostic tests to support community-based testing for priority medical conditions across three rural and peri-urban communities in Guinea. An existing web-based application was modified to include integrated symptom screening for malaria and COVID-19, maximize workflow efficiency and conduct end-to-end data capture on tablet devices. Using the application, community health workers screened participants for symptoms of malaria and COVID-19, with eligible participants tested using rapid diagnostic tests. All participants also underwent blood pressure and blood glucose measurements, while malnutrition screening was offered to pregnant women or children under 5 years. Services were provided to residents through mass consultations and home care visits across the study locations. The intervention reached 5204 people overall, with 3241 people enrolled via the application. 32.4% and 15.8% of participants had elevated blood pressure and blood glucose levels, the majority of whom were previously undiagnosed. Of those tested for malaria, 3.2% (*n* = 28/876) tested positive. The digital tool was successful in providing end-to-end data capture, with 99% of participants having their rapid diagnostic test results captured in real-time, and all outcomes reported into the Ministry of Health database. Together, the study demonstrates the feasibility of using a web-based digital tool to support community health workers with providing community-based diagnostic services in rural and peri-urban communities in a low-resource setting.

## INTRODUCTION

Guinea is a lower middle-income country in West Africa, and has a fast-growing population of ~14.2 million people [1]. An estimated 50% of Guineans lack adequate access to health facilities in close proximity to their home [2]. Close to half of the population (44%) lives in poverty [2], with the majority situated in rural areas, where there is heavy reliance on health posts and health centres that are often poorly equipped to provide essential services, and high out-of-pocket expenditure by patients [3].

Guinea experiences a high burden of communicable diseases, in particular malaria, which is a leading cause of illness and death in the country especially in children under 5 years, and is the most frequent reason for hospitalizations [4, 5], placing a strain on the country’s already-limited health infrastructure. Malnutrition rates are high, with 6.1% of children under 5 years affected by global acute malnutrition [6]. Guinea also faces high prevalence rates of noncommunicable diseases, particularly hypertension and diabetes, for which adequate diagnosis and treatment are not readily available at primary care level, especially in rural areas where rates of hypertension are higher [7, 8]. The COVID-19 pandemic and resurgence of Ebola in 2021 have also stretched the country’s health system and workforce in recent years [9]. Public health facilities in Guinea often face a chronic shortage of human health resources, particularly physicians, nurses, lab technicians and pharmacists, as well as insufficient diagnostic capacity for basic tests such as COVID-19, blood glucose tests, electrocardiograms (ECGs) and X-rays. There is also a disparity in the distribution of health workers between rural and urban areas, whereby rural settings have fewer health workers than urban ones [10, 11]. As a result, people in particularly rural areas often travel long distances to access basic diagnostic services, which is costly both in time and money.

In recent years, there has been growing interest in utilizing digital tools to improve access to medical services, particularly in remote and resource-constrained settings. Digital tools, such as mobile and tablet applications, can support the delivery of healthcare outside of traditional settings, by providing a way for patient medical information and test results to be accurately recorded and transmitted in the field [12–15]. Digital tools can also include decision-support tools (such as screening algorithms) to standardize testing approaches and provide real-time clinical guidance to health workers, especially those with less advanced clinical training, who can be monitored and supported remotely. They also facilitate the linkage of patients in the community to the broader health system for further care, for example by allowing more efficient transmission of patient data and enabling follow-up by mobile phone, something which is often challenging for people living in rural areas [12–15].

In this context, Clinic+O was established in September 2020 as a Guinean non-governmental organization to facilitate healthcare access through the use of digital technology to support active outreach services. Through the active outreach services, testing and treatment for diabetes and hypertension are delivered at the community level, with the digital platform providing an electronic medical record to enable continuity in care for all patients. Working with community health workers (CHWs), local doctors and nurses, alongside a network of local pharmacies and laboratories, Clinic+O has expanded access to patient-centred primary care for marginalized populations who may otherwise not access basic health services, such as screening for high blood pressure, diabetes and childhood malnutrition. This work has been undertaken in collaboration with Guinea’s Department of Community Health within the Ministry of Health, which envisions building a nationwide network of CHWs who are digitally trained and equipped with tools that facilitate accurate data collection at the local level.

In 2023, Clinic+O, in collaboration with Guinea’s Ministry of Health and the global non-profit FIND, undertook a project to provide additional community-based testing, for malaria, COVID-19 and malnutrition, alongside Clinic+O’s existing package of diabetes and hypertension screening. The project expanded Clinic+O’s existing digital system to create an optimized workflow that included an integrated symptom screening algorithm for malaria and COVID-19, which used digital logic to identify individuals in need of rapid testing based on responses captured by the CHW. The optimized workflow also provided automatic interpretation of clinical data for key parameters, such as indicators of malnutrition, so as to flag individuals in need of further care, and supported referral to the nearest health centre, as well as patient-follow up.

The project was designed and implemented in close collaboration with community leaders and the Ministry of Health. Key stakeholders in the project were identified and engaged ahead of the project, including mayors, district presidents, sector heads, young people, women, imams, health centre leads and CHWs. Clinic+O’s vision and the project aim were presented to key stakeholders in a series of meetings held in community spaces.

Several working sessions were conducted to obtain feedback from key stakeholders around the design of the application and to determine clinical best practice. Field trips were undertaken to observe how CHWs and patients interacted with the existing web-based application, to inform the adaption of the workflow to incorporate testing for additional diseases. Best practice pathways were also created for processes that did not previously exist in Guinea, based on discussions with clinicians and adaptation of World Health Organization guidelines. A 1-month pilot was also conducted in Mamou, a rural area of Guinea where Clinic+O has ongoing community activities, to test the clinical and digital workflows in the field and identify areas for further optimization before moving to wider implementation. In the pilot phase, a total of 1327 participants were enrolled, the majority (*N* = 1294; 97.5%) of whom completed the workflow. Out of the 208 people tested for malaria, 21 people (10%) tested positive for malaria (15 were males and 6 were females). Around 20% (243/1218) had high blood pressure (138 were males and 105 were females), the majority of whom weren’t previously diagnosed with hypertension. A further 11.2% (139/1241) had high blood glucose (65 were males and 74 were females). Only 0.6% people tested positive for COVID-19 (1/156) and one test was inconclusive.

Following the pilot, this study assessed the feasibility of deploying the tool as part of digitally supported community screening in Guinea. The study was conducted in three sites in Guinea: (i) Coyah, a rural area; (ii) Dubréka, a peri-urban town located on the north side of Conakry; and (iii) Soumambossia, a low-income peri-urban district of Conakry comprised of densely populated slums. Coyah and Dubréka are in lower Guinea and are ~65 km away from Conakry, the capital city. Coyah has a population of 281 757 inhabitants with 1 hospital and 6 community health centres, while Dubréka has a larger population of 352 859 people with 1 hospital and 10 community health centres. Soumambossia has a population of 25 000 people with only one community health centre [16]. All these areas are characterized by a high prevalence of communicable and noncommunicable diseases, including diabetes and hypertension, particularly among the most vulnerable groups, including pregnant women, children and the elderly. Communities in all three locations lack access to primary care services due to inadequate medical infrastructure, high cost of primary care and long-distance travel to medical facilities. Most community members are farmers who make an average daily income of less than US $2. For most low-income people in rural communities, the high cost of transportation (US $22+) to travel to Conakry alone, along with hospital-related administrative fees and lodging is a significant barrier to seeking treatment, whether for minor or major health-related conditions. People from Coyah, Dubréka and Soumambossia seeking care at hospitals in Conakry spend considerable time travelling to receive healthcare, as the average round trip to a hospital in Conakry takes ~2 to 4 hours. The long travel time creates challenges for patients and their families, including the loss of daily income, lack of food for the day, increased exposure to diseases, and difficulties finding transportation back home. Many people living in the study areas walk to local community health centres, which is time-consuming and challenging.

Consequently, this project set out to reduce the barrier of long-distance travel, transportation costs and inadequate medical infrastructure that prevents low-income patients in these areas from accessing primary care, by deploying digitally supported community screening for the previously mentioned priority conditions.

Here, we share findings around the implementation, feasibility, and acceptability of the community-based, digitally supported screening model across three rural and peri-urban communities in Guinea.

### Study objectives

The primary objective of the study was to evaluate the feasibility of deploying integrated COVID-19 and malaria symptom screening and diagnosis by CHWs, using digital tools for decision support and end-to-end data capture, and point-of-care screening tools for COVID-19, malaria, blood pressure, blood glucose levels and malnutrition. The secondary objectives of this study were to:

- measure the proportion of the study population with conditions being tested (COVID-19, malaria, elevated blood pressure and elevated blood glucose levels);
- measure the proportion of children <5 years and pregnant women with signs of malnutrition;
- provide linkage to care for all eligible participants; and
- assess the acceptability of intervention in the study population.

The qualitative experiences of CHW using the tools were also assessed as an exploratory endpoint.

## MATERIALS AND METHODS

### Study design and population

This was an interventional operational research study of community-based rapid testing of malaria and COVID-19 by CHWs using a digital tool for integrated decision support and end-to-end data capture in Guinea. The study also offered screening for noncommunicable diseases (blood pressure and blood glucose levels) to all participants and malnutrition screening for pregnant women and children under 5 years. Eligible participants were adults and children in the target communities who provided consent.

### Digital tool and workflow design

The modification of the digital tool was necessary before assessing the feasibility of the digital tool for providing real-time decision support and end-to-end data capture in community-based testing. Specifically, the application was modified to enable the registration of demographic data and patient medical history, and enable the recording of vital parameters such as oxygen level and body temperature, and symptoms such as fever, cough and muscle pain. An algorithm was also developed combining oxygen level, body temperature and symptoms to aid in the decision-making process, i.e. whether the patient should take a rapid diagnostic test (RDT) for suspected conditions (malaria and COVID-19, malaria only, or COVID-19 only)—based on national clinical guidelines in consultation with the Ministry of Health.

The workflow for the COVID-19 and malaria RDTs also integrated a timer to ensure timely reading of the RDT result in line with manufacturers’ instructions. The application also prompted blood glucose and blood pressure measurements for all eligible individuals, with all results captured in their record. Additionally, children under 5 years and pregnant women were prompted to undergo malnutrition screening, based on the middle upper arm circumference (MUAC) measurements with inbuilt digital logic automatically flagging those at risk of malnutrition based on Ministry of Health guidance on MUAC parameters.

The application was also modified to guide the distribution of medication on site for participants who tested positive for malaria based on standard dosing guidelines and referrals for participants who need further medical attention. A unique identifier generated by the system was used by focal points at receiving health facilities to track which referred patients reported for care, with individuals who did not visit the facility followed up via phone call.

### Stakeholder engagement

In collaboration with the local Ministry of Health and the heads of community health centres, 45 CHWs (36 females and 9 males) were recruited and trained for the project. However, only 30 CHWs (24 females and 6 males) were subsequently involved in the project: 15 of whom worked for the government and 15 of whom were independent CHWs and 15 CHW were backups.

Awareness of the project was raised by authorities, as well as the FIND and Clinic+O teams. Local authorities were involved in informing and mobilizing community members, raising awareness of the benefits of the services offered through information-sharing in places of worship and markets, and logistical support (e.g. provision of chairs, tables). The FIND and Clinic+O teams supported CHWs to raise awareness of Clinic+O services in households and at weekly markets in Coyah, Dubréka and Soumambossia.

### Training

The 45 CHWs were trained over 3 days on the use of electronic medical devices and rapid testing to facilitate accurate screening and diagnosis. CHWs learned how to use the medical kit for the conditions covered in the study, including an electronic blood pressure monitor, a blood glucose meter, an oximeter, a thermometer, RDTs and upper arm circumference measuring tape to assess malnutrition risk among both adults and children.

Following the medical training, CHWs were trained on how to use Clinic+O’s web application to deliver primary care services using tablet devices. Each CHW was equipped with a smart tablet and internet data to facilitate patient enrolment and trained on how to enter: (i) patient demographic information; (ii) patient vital information; (iii) patient medical history and patient photos to create medical ID cards and to capture the RDT results; and (iv) patient referral and follow up.

Training was also provided to 50 community leaders (15 men and 35 women) across the three project sites. Participants included 10 district leaders, 6 community leaders, 4 religious leaders, 3 heads of community health centres, 8 youth leaders, 5 local influencers and 14 administrative leaders. The training sessions enabled Clinic+O to build trust with local communities by showing participants the benefits of community-based testing services and of using digital tools to support quality healthcare. Local authorities involved in the project were also trained on the value of digital health technologies and the benefits of the early detection of illnesses.

### Study process

**Figure 1:**
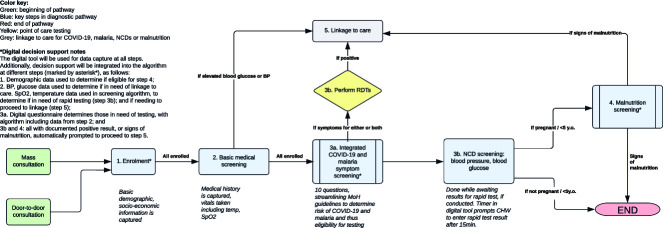
Community-based testing model workflow^*^. BP, blood pressure; CHW, community health worker; MoH, Ministry of Health; NCD, noncommunicable disease; RDT, rapid diagnostic test; SPO2, oxygen saturation level; y.o., years old. ^*^ Biowaste was placed into containers and disposed of at the bin of the nearest community health centre.

The community-based testing model workflow is outlined in Fig. 1. CHWs provided services through mass consultations and home care visits across the three study locations (Soumambossia, Coyah and Dubréka).

Mass consultations were offered by Clinic+O’s medical team, composed of nurses and CHWs equipped with a mobile van, tents and medical equipment. The mass consultations were strategically located and timed across the three sites to maximize access to the intervention and organized in collaboration with local and religious leaders to ensure high attendance.

The home care visits were door-to-door consultations conducted by CHWs to either enrol new patients or follow-up with existing patients enrolled in mass consultations. In addition to following up with enrolled patients, CHWs screened and enrolled other family members who were present during home care visits, and sought to enrol neighbours.

Participants enrolled during mass consultations and home care visits received a medical ID card and a digital medical record that enabled them to access the range of screening services offered during the study period. Individuals aged ≥18 years provided written consent to participate in the study and parents/caregivers provided written consent for children to participate. At enrolment, basic demographic and socioeconomic data were captured to determine eligibility for malnutrition screening. Pregnancy was self-reported by participants.

Once enrolled, participants had their medical history recorded in the Clinic+O app, and vitals were measured, including temperature, and oxygen saturation level. Participants were subsequently screened for symptoms of COVID-19 and malaria and eligibility for testing by RDT, using an integrated 10-question digital screening form focused on symptoms associated with the two conditions. This digital screening form was developed as a streamlined version of the Ministry of Health guidelines; questions are shown in Table S1.

If eligible, patients received RDTs for malaria and/or COVID-19. Participants were tested for COVID-19 using the Das Labor Biodiagnostic test and for malaria using the Abbott Bioline RDT, with both tests conducted according to manufacturer’s instructions.

A timer in the digital tool prompted the CHWs to enter the RDT results after 15 min. While awaiting the results of the RDT, participants were digitally screened for the noncommunicable diseases and their blood pressure and blood glucose levels were measured by the CHWs, as indictors for hypertension and diabetes, respectively. Pregnant women and children under 5 years of age were not screened for blood pressure and blood glucose, but were screened for malnutrition by measuring MUAC [17].

Participants testing positive on the COVID-19 RDTs, who had elevated blood glucose or blood pressure levels, and those with symptoms of malnutrition were linked to care (referred to the nearest health centre, or higher-level facility depending on the severity of their condition). However, participants testing positive on malaria RDTs were counselled and initiated on malaria treatment onsite by the CHWs.

Participants who were referred received a referral form containing their unique identifiers, which was recorded by a focal point at the receiving health facility. The focal was thus able to track participants who reported for care. By providing their unique identifiers to Clinic+O, it was possible for the team to identify those who had not visited the facility and follow up with them telephonically to encourage linkage to care and understand potential barriers to them visiting the health facility.

### Outcome measures

For the primary objective, the feasibility of the digital tool for providing real-time decision support and end-to-end data capture in the community was assessed through recording the proportion of records across the workflow that could be completed in real-time and the proportion of RDT results for which the web-based application was able to collect and store in real-time. The feasibility of deploying integrated COVID-19 and malaria symptom screening and diagnosis by CHWs using RDTs and the digital tools was further assessed through the number of participants who were successfully screened using the approach by target condition.

The secondary objectives were assessed as follows. The proportion of the study population with the conditions being tested was assessed based on the numbers identified during screening, along with the number of eligible participants linked to care. Participants were also asked to participate in a questionnaire about their daily income and willingness to pay for the services, to assess the acceptability of the intervention if a fee was charged for sustainability (for the purposes all the study, all services were provided free of charge).

Finally, the motivations and views of the CHWs on delivering digitally supported community-based testing were qualitatively assessed through structured questionnaires and focus-group discussions (exploratory objective).

### Sample size and analysis

The study had a feasibility-based target sample size of 3000 participants to be tested with RDTs. The sample size was calculated based on the number of people that could be feasibly screened within the study period and available study resources. Data were collected using the MySQL data management tool PHP Admin to retrieve the data in CSV format. The CSV data were structured into five key tables: demographic information, medical background, symptoms, vital parameters and RDT type. Data were validated, by checking for potential errors and inconsistencies, and cleaned. During the data cleaning process, duplicate entries were removed, and the dataset was examined for missing values.

The majority of participants had complete data, but for those with missing demographic and medical background information, the CHWs responsible for the participant in question were identified and asked to reach out to the participant via telephone to collect the missing information and submit it retrospectively via the web application. In some cases, CHWs also reported that internet connection issues prevented them from entering test results in real-time and in these cases they had to record the results in on paper. Consequently, the CHWs were asked to retrospectively update the application with this information from their paper records. The data tables were subsequently combined into one end-to-end dataset for analysis. Data were visualized and analysed using Google Data Studio and Dataiku DSS.

The project also set up a mechanism for Clinic+O to report data manually through anonymized spreadsheets uploaded into the country’s District Health Information Software 2 (DHIS2) under the credential of the community health centres of Coyah, Dubréka and Soumambossia. DHIS2 is an open source, web-based platform commonly used as a health management information system, including by the Guinean Ministry of Health.

### Ethical considerations

The study, including the pilot phase, received ethical clearance and approval from Comité National d’Ethique pour la Recherche en Santé (CNERS). Participants had the right to withdraw from the study at any time. The study was conducted in accordance with ICH GCP E6/R2 guidelines [18] and the Declaration of Helsinki [19]. Confidentiality was ensured by deidentifying raw data for the study team and restricting patient details to only the Ministry of Health in Guinea and the Clinic+O team that was responsible for direct provision of services.

## RESULTS

### Enrolment

Enrolment took place during 11 weeks between 6 April and 21 June 2023. A total of 21 mass consultations were conducted, which targeted an average of 100 participants, along with 16 home care visits by the CHWs. Overall, the study reached 5204 people: 2884 via the 21 mass consultations and 2320 via 16 home care visits.

Among the 5204 people reached via the mass consultations and home care visits, 3241 people were enrolled and had their data captured via the web application. The remainder of the people reached were sensitized on the study (i.e. provided with information) but not enrolled, primarily as they did not perceive an immediate need for health services. Across the mass consultations, 1701 people were enrolled overall: 599 participants across 8 mass consultations in Soumambossia (the district with the highest population density), 492 people across 9 mass consultations in Coyah and 610 participants across 6 mass consultations in Dubréka. In the home visits, 1540 people were enrolled from 941 homes across the three sites. The demographics of the study participants, disaggregated by gender, are shown in Table 1.

**Table 1:** Demographic characteristics of study participants

	**Male, *n* (%)**	**Female, *n* (%)**	**Total, *n* (%)**
**Sex**	1320 (40.7)	1921 (59.3)	3241 (100)
			
**Age (years)**			
0–5	177 (48.6)	187 (51.4)	364 (11.2)
6–12	278 (47.4)	308 (52.6)	586 (18.1)
13–17	109 (36.3)	191 (63.7)	300 (9.3)
18–24	157 (39.1)	245 (60.9)	402 (12.4)
25–34	175 (32.6)	361 (67.4)	536 (16.5)
35–44	154 (37.8)	253 (62.2)	407 (12.6)
45–54	103 (40.9)	149 (59.1)	252 (7.8)
55–64	73 (35.8)	131 (64.2)	204 (6.3)
65+	94 (49.7)	95 (50.3)	189 (5.8)
Blank	0	1 (100)	1 (0)
			
**Travel time to nearest facility (hours)**			
<1	785 (40.2)	1169 (59.8)	1954 (60.3)
1–2	514 (41.4)	727 (58.6)	1241 (38.3)
<2	19 (44.2)	24 (55.8)	43 (1.3)
2–4	2 (100)	0 (0)	2 (0.1)
4–8	0 (0)	1 (100)	1 (0)
			
**Highest level of education**			
University	194 (68.8)	88 (31.2)	282 (8.7)
High school	157 (65.1)	84 (34.9)	241 (7.4)
Junior high school	123 (39.8)	186 (60.2)	309 (9.5)
Elementary school	476 (44.2)	601 (55.8)	1077 (33.2)
Non-Western education^a^	370 (27.8)	962 (72.2)	1332 (41.1)

a Participants attended Madrassa and can read and write in Arabic script

### Feasibility of deploying digitally enabled integrated screening

#### Assessment of digital tool feasibility for providing real-time decision support and end-to-end data capture in community-based testing

Overall, the workflow process could be completed from end-to-end for 98% (*n* = 3168) of participants. The most common reason for CHWs not completing the workflow from end-to-end was poor internet connection in some home care visits, particularly in very remote locations.

Nearly all participants (99%, *n* = 1871) had their RDT results for COVID-19 and malaria entered in real-time (i.e. as soon as the results were available). In a few cases, slow internet connection prevented CHWs from entering data into the application, and as a result 93 people (5%) had their RDT result missing for both COVID-19 and malaria. In these cases, CHWs collected the RDT data on a piece of paper and then entered it manually into the application at a later time.

For the screening of noncommunicable diseases, all participants who received blood glucose screening (*n* = 2941) and blood pressure results screening (*n* = 1982) had their results captured in the application.

#### COVID-19 and malaria testing

**Figure 2:**
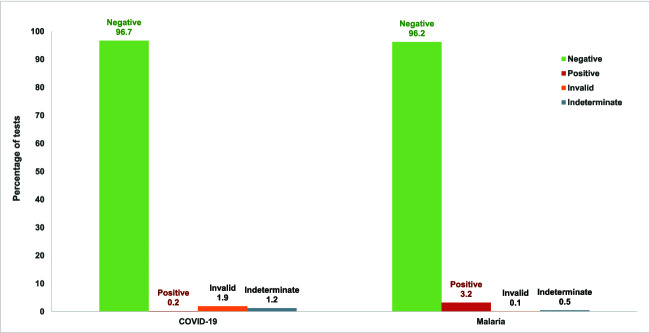
Proportion of participants testing positive for COVID-19 and malaria

All participants enrolled in the study were administered the integrated COVID-19 and malaria symptom screening using the digital tool (Table S1). Among those eligible, overall, 876 people (307 male and 569 female) were tested for malaria using the malaria RDTs. Of these, 28 people (3.2%; 16 females and 12 males) tested positive for malaria across the three locations (Fig. 2). Coyah had the highest number of positive cases (13) followed by Dubréka (10) and then Soumambossia (6). Overall, four tests (0.5%) produced indeterminate results and one test (0.1%) produced an invalid result. Among participants testing positive for malaria and tested for blood glucose levels, 5 (18%) had an elevated blood glucose. Malaria treatment was offered at the point of testing to all participants who tested positive for malaria.

**Figure 3:**
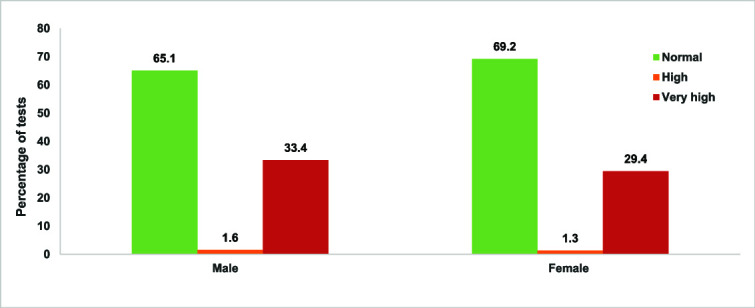
Participant blood pressure measurements by category and sex

Nine hundred and ninety-five participants had symptoms and were tested for COVID-19 (359 male and 636 female). Of these, only two people (0.2%, one male and one female), tested positive for COVID-19 (Fig. 2), reflecting the broader low COVID-19 positivity rates in Guinea at the time. One of the participants testing positive for COVID-19 also tested positive for malaria. The two participants who tested positive had mild symptoms and were referred to the community health centres of Dubréka and Soumambossia where they received proper guidance and support. The Clinic+O medical team also conducted a follow up call with these participants a week after their diagnosis. Both participants reported that they were doing fine and thus no further care was required.

#### Blood pressure screening

A total of 1982 participants were screened for elevated blood pressure (773 males, 1209 females). Among those screened, 1.4% of participants had high blood pressure, defined as systolic blood pressure over 130 (0.6% of males, 0.8% of females) and 31% of participants had very high blood pressure, defined as systolic pressure over 140 mmHg (13% of males, 18% of females) (Fig. 3). A further 13.4% of participants had previously been diagnosed with high blood pressure and were not screened as their status was known and they were already on treatment. Among participants with elevated blood pressure levels (*n* = 642), almost all (*n* = 614, 96%) had very high levels.

#### Blood glucose screening

**Figure 4:**
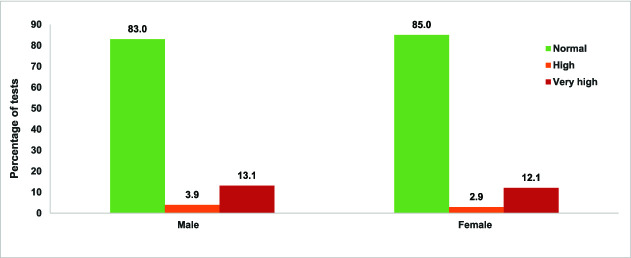
Participant blood pressure measurements by category and sex

Overall, 2941 participants were screened for blood glucose levels (1199 male, 1742 female). Of those tested, 3.3% had high blood glucose levels (1.5% of males, 1.7% of females) and 12.4% had very high blood glucose levels (5.3% of males, 7% of females) (Fig. 4). Around 3.5% of participants had previously been diagnosed with high blood glucose levels, so were not screened as their status was known and they were already on treatment for diabetes. Among participants with elevated blood glucose levels (*n* = 464), around half (*n* = 366, 55%) had very high levels. Screening also identified 154 (16%) of people who had both very high blood pressure and very high glucose levels.

#### Malnutrition screening

All pregnant women and children under 5 years were screened for malnutrition. Overall, 302 participants were screened for malnutrition by CHWs, with 260 children under 5 years and 41 pregnant women aged between 19 and 25 years screened. Among those screened, 45 (14.9%) had signs of malnutrition based on the MUAC measurement so were further assessed for malnutrition, and of these 15 (33.3%) were newly identified as having malnutrition.

#### Medical facility referral and follow-up

**Figure 5:**
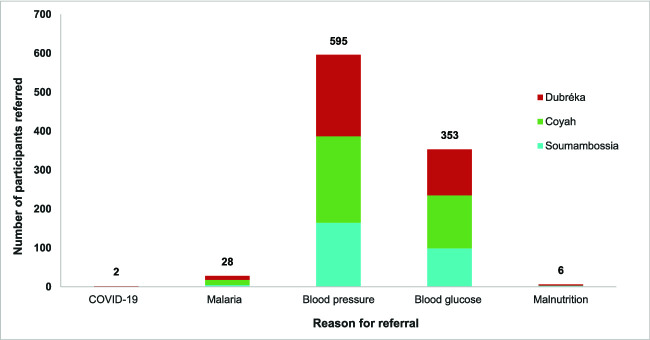
Participant referral to community health centre by condition and location

Throughout the study, 767 participants (322 male and 445 female) were flagged for referral to community health centres based on the Clinic+O algorithm and their screening results (reasons provided in Fig. 5). Participants were also referred if they had their blood glucose test conducted after eating breakfast and would therefore require an additional fasting blood glucose test to correctly identify whether they had elevated blood glucose due to diabetes. Participants who were on treatment for diabetes and/or hypertension were not referred to community health centres to avoid unnecessary trips for conditions already known to the participant and their medical practitioner.

Overall, 256 people received referral forms. Among the referred people, 64 people attended community health centres overall, 32 in Coyah and 29 in Dubréka. No participants attended the community health centre of Soumambossia. Of the 64 participants who attended, 17 were referred for high blood glucose, and 47 for high blood pressure. It was not possible to routinely determine whether people who had been referred visited other private health facilities that were not being tracked in the study.

Out of those referred, 64 people (25%) received follow up calls, among which 68% (*n* = 52) reported attending medical facilities. The other 32% (*n* = 24) of people reported not attending the medical facilities, for reasons including lack of time to go to the medical facility and a lack of transportation.

### Acceptability of the intervention in the study population

All participants who enrolled in the study were asked about their willingness to pay for the services they received, based on Clinic+O’s envisioned subscription model that would entail participants paying an annual fee for access to regular screening and treatment services when needed. 98% (*n* = 3189) reported that they were willing to pay between US $10 and $12, while 1.7% were willing to pay between $20 to $30. Only 17 people (0.5%) were unwilling to pay for the services.

Participants were also asked about their daily income and among respondents (*n* = 2041), 16% reported that they earn less than US $2 a day, 64% reported that they make between US$ 2–4 a day and 17% reported that they make US$ 4–6 a day. A further 2% said that they make US$ 6–10/day and 0.5% said that they make more than US$ 10/day.

### **Qualitative assessment of** CHW **experiences**

Thirty CHWs were interviewed in a focus group meeting to gain insights into their motivations to participate in the project and experiences conducting the intervention. The CHWs were aged 23 to 31 years old and 80% were female. All CHWs had a junior high school education and were recruited in collaboration with local leaders and local representatives of the Ministry of Health.

All CHWs reported that they were motivated by the opportunity to learn how to use digital devices such as tablets and web-based applications to offer health services. Two-thirds of the CHWs reported that they took part as they wanted to acquire medical and digital skills that they could use to offer primary care services. Another third of CHWs noted that they wanted to belong to a modern organization that offers quality care to low-income communities.

Half of the CHWs mentioned that prior to the study, they had limited medical knowledge of collecting patient vital information such as blood pressure and blood glucose levels. However, all the CHWs reported knowing how to correctly perform and interpret the result of malaria and COVID-19 RDTs and measure MUAC to assess malnutrition.

All CHWs reported that this was their first time using an application to enrol patients, collect data and assist with providing care. Furthermore, all CHWs shared that this was their first time using an application with an embedded screening algorithm to inform their decision-making. CHWs mentioned the screening algorithm helped reduce medical errors and facilitated the gathering of patients’ demographic, vital and medical information. Filling out the digital form was quick, with CHWs reporting a completion time of 5–6 min, with internet connection. Additionally, incorporating an RDT for either COVID-19 or malaria during screening took about 15 min; CHWs were allowed to continue with the screening process while waiting for the RDT results. CHWs also shared that the user interface was easy to use as it has a step-by-step logic inbuilt with a symptom-based screening model. Two thirds of CHWs suggested building an offline version of the application to mitigate the internet challenges that come with offering services in locations that have limited internet services.

All CHWs reported that they were now confident in their ability to provide diagnostics and highlighted that when they master the skills to provide essential services, patients will be able to access care without having to travel. However, all CHWs also said that they see risks associated with provision of diagnostic services: most (*n* = 28/30, 93%) mentioned that the main risk is contamination through exposure to biological fluids such as blood, saliva and exposure to other potential infections. Seven CHWs also noted the risk of errors or misinterpreting results if CHWs have gaps in their skillset.

All CHWs believed that they have an important role to play in diagnostics, highlighting that their work enables patients in remote areas to know their health status. The CHWs also noted that their work can help increase health coverage in their communities as people have confidence in their abilities. Similarly, all CHWs said that they received adequate training for their tasks in this project and would be keen to deepen their knowledge of the targeted pathologies, including high blood pressure and diabetes.

All CHWs said that they felt supported by the Clinic+O team and the local community leaders who have helped them sensitize the communities to the intervention. In addition, all CHWs reported that many people in the community appreciate their work and have encouraged them to continue offering services beyond the project.

## DISCUSSION

This study demonstrates the feasibility of utilizing a digital tool to support community-based screening for COVID-19, malaria and other priority medical conditions across rural and peri-urban communities in Guinea. In the study, CHWs were able to successfully conduct community-based screening using the customized digital application combined with RDTs for COVID-19 and malaria. The digital tool successfully provided real-time decision support and data capture, with 98% of workflows completed end-to-end and 99% of participants having their RDT results captured in real-time, which were then reported to the Ministry of Health through DHIS2. Although Clinic+O’s application was not integrated with DHIS2 during the study period, the success of the intervention prompted the Ministry of Health to request its integration. Currently, Clinic+O is developing a new version of the application, incorporating an API that will enable seamless linkage with DHIS2, facilitating enhanced data sharing and reporting.

Through the application, the project team was able to remotely monitor activities across all sites in real time, enabling early identification of challenges and provision of targeted support where needed. This ensured that quality, standardized clinical processes could be followed across disseminated sites of service provision, and CHWs with limited medical training could be supported to provide essential testing services to a population who would otherwise have faced significant access barriers. The digital tool also helped ensure that the CHWs and the rest of the medical team followed the workflow as designed, without missing any steps, ensuring interventions were recorded accurately. If errors were made, they were usually obvious and easily addressed. Digital data capture has been shown to reduce the risk of human error compared with paper-based reporting [20].

The malaria positivity rate in the study was 3.2%, which is lower than the national prevalence of 34% by RDT, as assessed in 2021 [2]. It is possible that the lower prevalence seen in our study is because more adults than children were recruited in this study and malaria is more prevalent in children than adults. Another plausible reason is that the study sites have a well-funded malaria programme, and community members have sufficient awareness that they can be diagnosed and treated for free at health facilities if they have symptoms of malaria. By contrast, in the rural pilot site of Mamou which has less advanced health services, a higher positivity rate of malaria (10%) had been found among 208 participants tested, and the number of malaria cases diagnosed through pilot exceeded the total number of malaria cases reported Mamou’s community health centre during the 1-month pilot period. Furthermore, the low positivity for malaria is possibly because the study took place during the dry season when malaria positivity is low. Overall, 0.2% of participants tested by RDT in the study were positive for COVID-19. The low COVID-19 positivity rate corresponds to the national COVID-19 rates at the time of the study, where only ~100 cases were reported across the entire country [21].

Our study also identified a notable proportion of participants with undiagnosed high blood pressure and blood glucose levels. Of participants with blood pressure assessments, 32.4% had high or very high blood pressure levels, 64.6% (*n* = 415/642) of whom did not have any previous diagnosis of high blood pressure. In addition, 15.8% of participants screened for blood glucose levels had high or very high levels, 68.5% (*n* = 318/464) of whom had no previous diabetes diagnosis. While these measurements do not indicate a definitive diagnosis of hypertension or diabetes, which required further investigation after referral, these findings revealed high rates of undiagnosed high blood pressure and blood glucose levels among these communities. High pressure and blood glucoses levels leave people at increased risk of cardiovascular complications and diabetes. Similarly, among the 45 people screened for malnutrition, 33.3% (*n* = 15) were identified as having malnutrition. The early identification of these conditions at the community level provides an opportunity for those affected to seek medical care, additional testing and treatment. However, there is a need to strengthen the post-referral pathway to ensure linkage to care, or to expand the services available at the community level, so that further clinical assessment and treatment can be provided onsite, where indicated by the initial screening.

One of the major innovations of this intervention was the integration of rapid testing with primary health services. Through rapid testing, CHWs were able to screen patients onsite, either in their homes or at the mass sites, and provide an accurate result for COVID-19 and malaria without requiring participants to travel to the community health centres, which could require additional travel time and transportation costs. Through the project, CHWs were also able to treat participants testing positive for malaria onsite.

Designing digital tools with intended users is an important component of the principles for digital development [22]. As such, it is important to understand the experiences and perceptions of the intervention by the community and CHWs. The intervention was found to be well received by both the community and the CHWs. Most participants (99.5%) interviewed were willing to pay for the services offered as part of the study. Participants also reported being open to using Clinic+O’s digital health solution to access primary care with the help of a CHW who has a tablet. In addition, when interviewed as part of a focus group meeting, CHWs reported an interest in continued use of digital devices and acquiring medical and digital skills to offer primary care services in the community.

The COVID-19 pandemic highlighted the importance of timely testing for the control of infectious diseases and highlighted the many gaps in diagnostics and primary care that leave people vulnerable to communicable and noncommunicable illnesses. While the pandemic placed immense pressure on health systems, it also spurred the development and application of innovative technologies and approaches to make testing more readily accessible. In particular, the widespread use of COVID-19 RDTs during the pandemic increased familiarity with rapid testing models. Several digitally supported RDT testing approaches have since been deployed to evaluate how digital tools can be used to overcome challenges of testing in the field. In South Africa, a testing intervention using a mobile-based digital tool combined with RDTs was successfully used to improve access to COVID-19 screening across taxi ranks in Johannesburg [15]. Similarly, digital health tools have been used to enhance COVID-19 and tuberculosis testing and linkage to care among Boda boda motorbike riders in Nairobi, Kenya, a population at high risk for both diseases [23]. These studies, and others, demonstrate how digital tools can improve information management, streamline workflows and improve patient care [24, 25]. The findings from this study add to the growing body of evidence on the feasibility and benefits of utilizing digital tools and RDTs to expand access to testing and primary care services in community settings.

In implementing the project, challenges included that some market vendors did not want to enrol in the study during business hours due to fear of losing income. To surmount this, vendors and other businesspeople should be enrolled for services outside working hours. Another challenge was internet access, particularly during home visits in rural areas, which prevented some CHWs from entering data into the web-based application in real-time. Although this solution was identified in the first phase of the project, time constraints prevented the creation of an offline version of the application. Consequently, developing an offline version of the application could be considered in the future to improve usability in areas with poor internet connection.

Altogether, the digital tool provided specific advantages for the implementation of community-based screening for priority diseases. In particular, through providing decision-support, the digital tool enabled screening and clinical-decision making by lower-cadre health workers. In addition, the digital tool also provides Guinea’s Ministry of Health with detailed information about the distribution of diseases in the study population, complemented by demographic information, which can inform future interventions. Based on the success of this intervention, Guinea’s Ministry of Health is planning to work with Clinic+O to integrate digitally enabled primary healthcare more broadly in Guinea so as to expand access to primary health services. This would include home care visits to assess and record patient baseline conditions and establishing a network of rapid testing sites to expand access to basic services for rural community members.

## CONCLUSION

In conclusion, this study demonstrates the feasibility of using a web-based digital tool to support screening for COVID-19, malaria and other priority medical conditions across rural and peri-urban communities. The study identified high rates of elevated blood pressure and blood glucose levels among participants across the study locations in Guinea, highlighting a need to increase screening and diagnostic testing among communities that have limited access to basic healthcare services. Together, the findings demonstrate the value of digitally enabled healthcare models that can increase access to testing and other essential healthcare services in underserved communities.

## Supplementary Material

OODH_2024_Supplement_15_10_2024_TU_oqae044

## Figures and Tables

**Table:** 

**Contributor Role**	**Role Definition**	**Author initials**
**Conceptualization**	Ideas; formulation or evolution of overarching research goals and aims.	N.D., P.A., M.K., M.C., N.B., A.B., K.S., R.K.
**Data Curation**	Management activities to annotate (produce metadata), scrub data and maintain research data (including software code, where it is necessary for interpreting the data itself) for initial use and later reuse.	A.D. and M.B.
**Formal Analysis**	Application of statistical, mathematical, computational or other formal techniques to analyse or synthesize study data.	A.D., K.S. and P.A.
**Funding Acquisition**	Acquisition of the financial support for the project leading to this publication.	R.K.
**Investigation**	Conducting a research and investigation process, specifically performing the experiments, or data/evidence collection.	N.D., A.K.B., M.B., A.D. and M.K.
**Methodology**	Development or design of methodology; creation of models	N.D., P.A., M.B., N.B. and A.D.
**Project Administration**	Management and coordination responsibility for the research activity planning and execution.	N.D., A.K.B., M.C. and M.K.
**Resources**	Provision of study materials, reagents, materials, patients, laboratory samples, animals, instrumentation, computing resources or other analysis tools.	N.D., A.K.B., A.D., M.C. and M.K.
**Software**	Programming, software development; designing computer programs; implementation of the computer code and supporting algorithms; testing of existing code components.	M.B. and N.B.
**Supervision**	Oversight and leadership responsibility for the research activity planning and execution, including mentorship external to the core team.	N.D., P.A., N.B., A.K.B., and K.S.
**Validation**	Verification, whether as a part of the activity or separate, of the overall replication/reproducibility of results/experiments and other research outputs.	P.A. and K.S.
**Visualization**	Preparation, creation and/or presentation of the published work, specifically visualization/data presentation.	N.D., A.D., A.K.B., A.B. and P.A.
**Writing—Original Draft Preparation**	Creation and/or presentation of the published work, specifically writing the initial draft (including substantive translation).	T.U. and P.A.
**Writing—Review & Editing**	Preparation, creation and/or presentation of the published work by those from the original research group, specifically critical review, commentary or revision – including pre- or post-publication stages.	N.D., P.A., K.S., A.K.B., A.D., A.B., N.B., and R.K.

## Data Availability

The data underlying this article will be shared on reasonable request to the corresponding author.

## References

[1] The World Bank. Guinea [accessed 3 November 2024]. Available from: https://data.worldbank.org/country/guinea

[2] U.S. President's Malaria Initiative. Guinea Malaria Profile, 2022 [accessed 3 November 2024]. https://d1u4sg1s9ptc4z.cloudfront.net/uploads/2023/01/Guinea-Malaria-Profile-1.pdf

[3] The World Bank. Poverty & Equity Brief: Guinea, 2020 [accessed 3 November 2024]. https://databankfiles.worldbank.org/public/ddpext_download/poverty/33EF03BB-9722-4AE2-ABC7-AA2972D68AFE/Global_POVEQ_GIN.pdf

[4] The World Bank. Guinea Health Service and Capacity Strengthening Project, 2018 [accessed 7 June 2024]. Available from: https://documents1.worldbank.org/curated/en/784191524880922883/pdf/GUINEA-REVISED-PAD-04062018.pdf

[5] World Health Organization. Top 10 Causes of DALY in Guinea for both Sexes Aged All Ages, 2019 [accessed 8 September 2023]. Available from: https://www.who.int/data/gho/data/themes/mortality-and-global-health-estimates/global-health-estimates-leading-causes-of-dalys

[6] World Food Programme. Guinea [accessed 8 September 2023]. Available from: https://www.wfp.org/countries/guinea#:∼:text=Although%20rich%20in%20natural%20resources,and%2012%20percent%20are%20underweight

[7] Kake A. Diabetes care in Guinea: challenges and solutions. *J Soc Health Diab* 2019;7:34–5

[8] Camara A, Toure A, Bangoura ST et al. Factors associated with knowledge of hypertension in rural Guinea, 2023: case of the rural commune of Maferinyah. *Eur J Prev Med* 2024;12:17–23

[9] UNICEF. Guinea Country Office Humanitarian Situation Report No. 1, 2021 [accessed 8 September 2023]. Available from: https://www.unicef.org/media/104211/file/%20Guinea-Humanitarian-SitRep-30-June-2021.pdf.

[10] World Health Organization. The World Health Report 2006: Working Together for Health, Geneva, Switzerland: World Health Organization, 2006 [accessed 19 June 2024]. Available from: https://www.who.int/publications/i/item/9241563176

[11] van de Pas R, Kolie D, Delamou A et al. Health workforce development and retention in Guinea: a policy analysis post-Ebola. *Hum Resour Health* 2019;17:63 31382972 10.1186/s12960-019-0400-6 PMC6683560

[12] Budd J, Miller BS, Manning EM, et al. Digital technologies in the public-health response to COVID-19. Nat Med 2020;26:1183–92. Epub 2020/08/10. PubMed PMID: 32770165 32770165 10.1038/s41591-020-1011-4

[13] Keutzer L, Wicha SG, Simonsson US. Mobile health apps for improvement of tuberculosis treatment: descriptive review. JMIR Mhealth Uhealth 2020;8:e17246. Epub 2020/04/22. 10.2196/17246. PubMed PMID: 32314977; PubMed Central PMCID: PMCPMC7201317 32314977 PMC7201317

[14] Davies C, Graffy R, Shandukani M, et al. Effectiveness of 24-h mobile reporting tool during a malaria outbreak in Mpumalanga Province, South Africa. Malar J 2019;18:45. Epub 2019/02/23. PubMed PMID: 30791909; PubMed Central PMCID: PMCPMC6385402 30791909 10.1186/s12936-019-2683-4 PMC6385402

[15] Majam M, Msolomba V, Venter F, et al. Monitored implementation of COVID-19 rapid antigen screening at taxi ranks in Johannesburg, South Africa. Diagnostics (Basel) 2022;12:402. Epub 2022/02/26. 10.3390/diagnostics12020402. PubMed PMID: 35204493; PubMed Central PMCID: PMCPMC8871379 PMC8871379 35204493

[16] Institut National de la Statistique. Institut National de la Statistique, 2023 [accessed 22 September 2023]. Available from: https://www.stat-guinee.org/#

[17] UNICEF. A Simple Tool to Detect Severe Malnutrition: The Mid-upper Arm Circumference (MUAC) Tape [accessed 8 September 2023]. Available from: https://www.unicef.org/supply/simple-tool-detect-severe-malnutrition-mid-upper-arm-circumference-muac-tape

[18] U.S. Department of Health and Human Services, Food and Drug Administration, Center for Drug Evaluation and Research (CDER), Center for Biologics Evaluation and Research (CBER). E6(R2) Good Clinical Practice: Integrated Addendum to ICH E6(R1) Guidance for Industry, 2018

[19] The World Medical Association I. Declaration of Helsinki, 2008 [accessed 3 November 2024].

[20] Walther B, Hossin S, Townend J, et al. Comparison of electronic data capture (EDC) with the standard data capture method for clinical trial data. PLoS One 2011;6:e25348. Epub 2011/10/04. 10.1371/journal.pone.0025348. PubMed PMID: 21966505; PubMed Central PMCID: PMCPMC3179496 21966505 PMC3179496

[21] World Health Organization. WHO Health Emergency Dashboard – Guinea, 2023 [accessed 8 September 2023]. Available from: https://covid19.who.int/region/afro/country/gn

[22] Development PfD. Design with People [accessed 7 June 2024]. Available from: https://digitalprinciples.org/principles/design-with-people/

[23] Karanja S, Aduda J, Thuo R et al. Utilization of digital tools to enhance COVID-19 and tuberculosis testing and linkage to care: a cross-sectional evaluation study among Bodaboda motorbike riders in the Nairobi Metropolis, Kenya. *PLoS One* 2023;18:e0290575. 10.1371/journal.pone.0290575 37682928 PMC10490987

[24] Wosny M, Strasser LM, Hastings J. Experience of health care professionals using digital tools in the hospital: qualitative systematic review. JMIR Hum Factors 2023;10:e50357. Epub 2023/10/17. 10.2196/50357. PubMed PMID: 37847535; PubMed Central PMCID: PMCPMC10618886 37847535 PMC10618886

[25] Alotaibi YK, Federico F. The impact of health information technology on patient safety. Saudi Med J 2017;38:1173–80. Epub 2017/12/07. PubMed PMID: 29209664; PubMed Central PMCID: PMCPMC5787626 29209664 10.15537/smj.2017.12.20631 PMC5787626

