# Supplementary material for: Feasibility and acceptability of a digital tool to support community-based screening for COVID-19 and other priority medical conditions across rural and peri-urban communities in Guinea
Source: Oxf Open Digit Health. 2024 Oct 25;2:oqae044. doi: 10.1093/oodh/oqae044 (PMC11932417; doi:10.1093/oodh/oqae044)
Supplement: OODH_2024_Supplement_15_10_2024_TU_oqae044 [file OODH_2024_Supplement_15_10_2024_TU_oqae044.docx]

**Supplementary materials**

**Feasibility and acceptability of a digital tool to support community-based screening for COVID-19 and other priority medical conditions across rural and peri‑urban communities in Guinea**

Nasser Diallo^1^, Mamadou Bobo^1^, Aboubacar Diallo^2^, Abdoul Karim Baldé^1^, Alpha Oumar Bah^1^, Mamady Kourouma^3^, Mamady Cisse ^3^, Nick Banks^4^, Rigveda Kadam^4^, Khairunisa Suleiman^4^ and Paula Akugizibwe^4^

^1^ Clinic+O, Commune de Ratoma, Conakry, Guinea

^2^ Independent Consultant, Johannesburg, South Africa

^3^ Department of Community Health, Ministry of Health, Guinea

^4^ FIND, Campus Biotech Chemin des Mines 9 1202, Geneva, Switzerland

***Corresponding author:** Nasser Diallo, Kaporo Rail, Commune de Ratoma, Guinea, (224) 627-32-07-07, [nasser@clinic-o.org](mailto:nasser@clinic-o.org)

**S1 Table. Symptom screening questions for COVID-19 and malaria**

| COVID-19 and Malaria Screening | | |
| --- | --- | --- |
| 1. Is there fever? | Automatically compute from vital parameters | All questions to feed into algorithm outlined in protocol: app will indicate whether individual should be tested for one, both or none of the conditions. |
| 1. Is there low blood oxygen? | Automatically compute from vital parameters |  |
| 1. Chills | Select: Y/N |  |
| 1. Nausea and vomiting | Select: Y/N |  |
| 1. Headaches | Select: Y/N |  |
| 1. Muscle or joint pain with no known cause | Select: Y/N |  |
| 1. Sore throat | Select: Y/N |  |
| 1. Cough | Select: Y/N |  |
| 1. Fatigue with no known cause | Select: Y/N |  |
| 1. Loss of sense of smell | Select: Y/N |  |
| 1. Difficulty breathing | Select: Y/N |  |
| 1. Diarrhea | Select: Y/N |  |
| 1. Does the patient need a malaria test? | Answer automatically pops up on screen based on responses | If they need a test, they will be directed to the instructions and timer |
| 1. What is the test result? | Select: positive/negative | Only ask if they did a test. |
| 1. Capture an image of the test | Take photo | Only ask if they did a test. |
| 1. Does the patient need a COVID-19 test? | Answer automatically pops up on screen based on responses | If they need a test, they will be directed to the instructions and timer |
| 1. What is the test result? | Select: positive/negative | Only ask if they did a test. |
| 1. Capture an image of the test | Take photo | Only ask if they did a test. |
